# Author Correction: Propensity of selecting mutant parasites for the antimalarial drug cabamiquine

**DOI:** 10.1038/s41467-023-41287-6

**Published:** 2023-09-06

**Authors:** Eva Stadler, Mohamed Maiga, Lukas Friedrich, Vandana Thathy, Claudia Demarta-Gatsi, Antoine Dara, Fanta Sogore, Josefine Striepen, Claude Oeuvray, Abdoulaye A. Djimdé, Marcus C. S. Lee, Laurent Dembélé, David A. Fidock, David S. Khoury, Thomas Spangenberg

**Affiliations:** 1 The Kirby Institute, UNSW Sydney, Kensington, NSW 2052 Australia; 2 Université des Sciences, des Techniques et des Technologies de Bamako (USTTB), Faculté de Pharmacie, Malaria Research and Training Center (MRTC), Point G, PB1805 Bamako, Mali; 3 Medicinal Chemistry & Drug Design Global Research & Development, Discovery Technologies, Merck Healthcare, 64293 Darmstadt, Germany; 4 Department of Microbiology and Immunology, Columbia University Irving Medical Center, New York, NY 10032 USA; 5 Center for Malaria Therapeutics and Antimicrobial Resistance, Columbia University Irving Medical Center, New York, NY 10032 USA; 6 Global Health Institute of Merck, Ares Trading S.A., (an affiliate of Merck KGaA, Darmstadt, Germany), 1262 Eysins, Switzerland; 7 Wellcome Sanger Institute, Wellcome Genome Campus, CB10 1SA Hinxton, UK; 8 Biological Chemistry and Drug Discovery, School of Life Sciences, University of Dundee, DD1 4HN Scotland, UK; 9 Division of Infectious Diseases, Department of Medicine, Columbia University Irving Medical Center, New York, NY 10032 USA; 10 Present Address: Weill Cornell Medical College, New York, NY 10021 USA

**Keywords:** Drug development, Computational science, Parasite evolution

Correction to: *Nature Communications* 10.1038/s41467-023-40974-8, published online 25 August 2023

In this article, Vandana Thathy should have been denoted as an equally contributing author.

In addition, the Present address ‘Weill Cornell Medical College, New York, NY 10021, USA’ for Josefine Striepen was missing.

The original article has been corrected.

