# Supplementary material for: Propensity of selecting mutant parasites for the antimalarial drug cabamiquine
Source: Nat Commun. 2023 Aug 25;14:5205. doi: 10.1038/s41467-023-40974-8 (PMC10457284; doi:10.1038/s41467-023-40974-8)
Supplement: Supplementary file 1 — Supplementary Information [file 41467_2023_40974_MOESM1_ESM.pdf]

Supporting Information

Propensity of selecting mutant parasites for the antimalarial drug cabamiquine

Eva Stadler, Mohamed Maiga, Lukas Friedrich, Vandana Thathy, Claudia Demarta-Gatsi, Antoine Dara, Fanta Sogore, Josefine Striepen, Claude Oeuvray, Abdoulaye A. Djimdé, Marcus C.S. Lee, Laurent Dembélé, David A. Fidock, David S. Khoury, Thomas Spangenberg

Supplementary Materials and Methods

Efficacy studies in *Plasmodium falciparum*-infected NOD/SCID/IL2rγnull (NSG) mice: Infections and treatment

Cabamiquine (succinate salt by Merck KGaA Darmstadt Germany [MSC2576186-B4]) was assessed for efficacy in the *P. falciparum* NSG mouse model essentially as described by Jimenez-Díaz *et al*[1]. Briefly, cabamiquine was formulated in 7% Tween80, 3% ethanol and administered at different doses of cabamiquine to cohorts of age-matched female immunodeficient NSG (NOD.Cg-*Prkdc*^{scid} *Il2rg*^{tm1Wjl}/SzJ) mice (Jackson Laboratory, Bar Harbor, ME). These mice had been previously engrafted with human red blood cells (RBCs) (generously provided by the blood bank in Zürich, Switzerland). Prior to compound treatment, mice were intravenously infected with 2×10^7 *P. falciparum* Pf3D7$^{0087/N9}$-infected RBCs (day 0)[2]. On day 3 after infection, mice, in groups of 2-5, were randomly allocated to cabamiquine treatment. Parasitemia was measured by microscopy. Chimerism was monitored by flow cytometry using an anti-murine erythrocyte TER119 monoclonal antibody (Pharmingen, San Diego, CA) and SYTO-16 and then analyzed via flow cytometry in serial blood samples (2 µL) collected every 2-3 days until the completion of the experiment. Parasite recrudescence was monitored via slide reading by microscopy of Giemsa-stained thin blood smears. Once parasitemia exceeded 0.5%, 100 µL of blood was collected and stored at –80°C for subsequent sequencing analysis. Thereafter, animals received a second treatment with the same dose as previously administered. Efficacy was again monitored by

microscopy. If recrudescence occurred again, new blood samples were collected and stored at –80°C.

In vivo studies conducted at the Swiss TPH, Basel, were approved by the veterinary authorities of the Canton Basel-Stadt (permit no. 2303) based on Swiss cantonal (Verordnung Veterinäramt Basel-Stadt) and national regulations (the Swiss animal protection law, Tierschutzgesetz)[3].

Measurement of concentration of drugs in blood

Cabamiquine levels were measured in whole blood to determine pharmacokinetic parameters in mice from the efficacy study. Peripheral blood samples (20 µL) were collected at different time points (1, 2, 4, 6, 24, 48, 72, 96, 168, and 216 h post treatment), mixed with 20 µL of Milli-Q water and immediately frozen on dry ice. Samples were stored at –80°C until analysis. Blood from control mice was used for bioanalysis calibration and QC purposes. For liquid chromatography with tandem mass spectrometry (LC-MS/MS) analysis, the frozen samples were thawed and treated with two volumes equivalent of acetonitrile containing the internal standard. After centrifugation, one volume equivalent of supernatant was diluted with one volume equivalent of water containing heptafluorobutyric acid. The extracts were analyzed using LC-MS/MS (quantification by HESI ionization in positive ion mode) performed at Swiss BioQuant AG (Switzerland). A noncompartmental analysis was performed to determine pharmacokinetic parameters using the Phoenix WinNonlin program (version 6.3).

Asexual blood-stage culture, genomic DNA extraction, and targeted *P. falciparum eEF2* gene sequencing of *P. falciparum* recrudescent infections in NSG mice (Fidock laboratory)

Cryopreserved asexual blood-stage parasites from recrudescent infections in NSG mice treated with cabamiquine were thawed and cultured in vitro at 2% hematocrit in human O^+ RBCs in RPMI-1640 media, supplemented with 25 mM HEPES (Fisher), 50 mg/L hypoxanthine (Sigma Aldrich), 2 mM L-glutamine (Cambridge Isotope Laboratories, Inc.), 0.21% sodium bicarbonate (Sigma Aldrich), 0.5% (wt/vol) AlbuMAXII (Invitrogen), 7.5% O^+ human serum and 10 µg/mL gentamicin (Fisher).

Cultures were propagated in tissue culture flasks gassed with a mixture of 5% O_2, 5% CO_2, and 90% N_2 and maintained at 37°C. The parental *P. falciparum* strain Pf3D7$^{0087/N9}$ that has been adapted to proliferate in the humanized NSG mice was cultured in parallel as the drug-sensitive control. For the extraction of genomic DNA, cultures were lysed in 0.2% saponin and washed twice with phosphate-buffered saline. Genomic DNA was extracted using the QIAamp DNA Blood Mini kit (Qiagen). The 2.5 kb *Pf*eEF2 gene (PF3D7_1451100) was PCR-amplified using flanking primers (Supplementary Table 12).[3] The PCR conditions were as follows: 95°C for 3 min, 45 cycles at 98°C for 20 s, 55°C for 30 s, and 68°C for 2.5 min, with a final extension of 3 min at 68°C. Agarose gel electrophoresis was used to confirm the PCR product size. Sanger sequencing of PCR products was performed by Genewiz Inc. using 10 sequencing primers covering the *Pf*eEF2 gene, in addition to the PCR primers (Supplementary Table 12). Sequences were aligned to wild-type (WT) *Pf*eEF2 from the 3D7 genome reference strain and analyzed using Geneious 9.1.8. Electropherograms were visually inspected to identify mixed sequences indicating multiple subpopulations.

In vitro drug susceptibility assays performed on culture-adapted recrudescent parasites from infected NSG mice (Fidock laboratory)

To define the drug EC_{50} and EC_{90} values of asexual blood stage parasites, predominantly ring-stage cultures at 0.3% parasitemia and 1% hematocrit were exposed for 72 h to a range of 10 concentrations of each drug that had been 2-fold serially diluted in duplicates along with drug-free controls in 96-well flat bottom polystyrene plates (Fisher). All assays were performed in serum-containing complete media. Serial dilutions of the drug were performed using a Tecan Freedom Evo 100 liquid handler and parasite dilutions were added manually. Parasite survival was assessed via flow cytometry on an Intellicyt iQue3 (Essen Bioscience) using 1× SYBR Green (Invitrogen) and 200 nM MitoTracker Deep Red FM (Invitrogen) as nuclear stain and vital dyes, respectively. EC_{50} and EC_{90} values were calculated using linear interpolation as means ± SEM from 3-10 independent experiments.

Fitness costs

To estimate the impact of resistance on the parasite growth rate, the fitness cost per generation was calculated using data reported by Baragana *et al.*[4], which described co-culture experiments of the cabamiquine-resistant lines with a green fluorescent protein (GFP)-tagged WT competitor. The ratio of each of the four test lines (WT, E134D, L755F, and Y186N) to the GFP-tagged competitor was first calculated on days 0 and day 14, equivalent to 7 generations (n). The calculation of fitness cost per generation was performed using the following equation: $R' = R((1-x)^n)$, where R' is the ratio of test:GFP competitor at the end of the assay, R is the ratio at day 0, n is the number of generations and x is the fitness cost.[5] As the GFP line is relatively slow growing, all lines had a relative fitness 'advantage'. The effective fitness cost of the cabamiquine-resistant mutations was derived by calculating the difference of the mutant lines compared with the WT value.

Human volunteer infection (VIS) study

The human volunteer infection study (VIS) data used were obtained from a randomized, single dose, Phase Ib clinical trial, using the induced blood-stage malaria (IBSM) model to characterize the pharmacokinetics and pharmacodynamics associated with the administration of cabamiquine. Briefly, healthy human volunteers were infected via intravenous injection of blood-stage *P. falciparum* 3D7 strain parasites on day 0. Parasite growth was monitored by collecting blood samples and performing quantitative PCR (qPCR) targeting the gene encoding 18S rRNA. The volunteers received a single oral dose of 150, 400, or 800 mg cabamiquine after 8 days[6], and clearance of parasitaemia was measured using qPCR. Antimalarial concentration in blood was determined at different time points after the administration. Artemether/lumefantrine (A/L) was administered in response to recrudescence of parasitemia or 20 days after cabamiquine dosing if recrudescence was not observed. The study was conducted at Q-Pharm Pty Ltd. (Brisbane, Australia) and approved by both the QIMR Berghofer and Australian Red Cross Blood Service Human Research Ethics Committees and were conducted in accordance with the Declaration of Helsinki. The study is registered with ClinicalTrials.gov (NCT03261401).

Estimation of the frequency of mutants from the data

We estimated the frequency of resistant mutants based on the data obtained from the different experiments using a limiting dilution assay-like approach. This method is a likelihood approach and assumes a binomial distribution of resistant mutants wherein success probability is the fraction of resistant mutants, and the sample size *n* is the inoculation size of a well. We assumed that if a well was positive, i.e. there was growth after treatment, then there was at least one resistant mutant in the inoculum and if it was negative, then there were no resistant mutants in the inoculum. Thus, the probability of no resistant mutants in a well with an inoculum of *n* parasites was calculated as follows:

$$P(0 \text{ resistant parasites} \mid n, f) = B(0|n, f), \tag{1}$$

where *B* denotes the binomial distribution, *f* is the fraction of resistant mutants, and the probability of at least one resistant mutant was calculated as follows:

$$P(\geq 1 \text{ resistant parasite} \mid n, f) = 1 - B(0|n, f). \tag{2}$$

The probability of *x* positive wells out of n_w wells (all with inoculum *n*) was calculated as follows:

$$\begin{aligned} P(x \text{ positive wells} \mid n_w, n, f) &= B\left(x \middle| n_w, P(\geq 1 \text{ resistant parasite} \mid n, f)\right) \\ &= B\left(x \middle| n_w, 1 - B(0|n, f)\right). \end{aligned} \tag{3}$$

Combining wells with different inocula, we obtained the following likelihood function for the frequency of resistant mutants:

$$\begin{aligned} L(f) &= \prod_{i=1}^{n_{in}} P\left(x_i \text{ positive wells for inoculum } i \mid n_{w,i}, n_i, f\right) \\ &= \prod_{i=1}^{n_{in}} B\left(x_i \middle| n_{w,i}, 1 - B(0|n_i, f)\right), \end{aligned} \tag{4}$$

where the subscript *i* indicates the *i*th inoculum, x_i is the number of positive wells, $n_{w,i}$ is the number of wells, and n_i is the inoculum size for the *i*th inoculum. We determined the maximum likelihood estimate for the fraction of resistant mutants by minimizing the negative log likelihood function using published data for the 3D7 strain or the Dd2 strains (Supplementary Table 2).

For the NSG and VIS datasets, we used the same approach to estimate the frequency of mutants. First, we computed the number of parasites at the time of treatment assuming a blood volume of 2 mL, a hematocrit level of 70%, and a mean human RBC volume of 90 fL for the NSG mouse data and a blood volume of 5 L for the VIS data. For the NSG data, we combined data regarding treatment with different cabamiquine doses (2 mice treated with 30 mg/kg and 9 mice treated with 12 mg/kg) and we assumed that 8 out of 11 mice had recrudescence with resistant parasites (based on genetic analysis of recrudescing parasites, 3 mice treated with 12 mg/kg had recrudescence with WT parasites). We then assumed that each mouse and human volunteer with recrudescence with resistant parasites had at least one resistant parasite at the time of treatment to estimate the fraction of resistant parasites as outlined above.

Of note, this approach assumed independence in the probabilities of resistance among parasites from within the same well/animal/individual compared with between individuals. This may be a simplification for the experimental systems wherein parasites multiply separately within different wells/animals/individuals prior to treatment and may thus have more relatedness within a well/animal/individual, than between individuals. A more sophisticated method may be possible, but it requires consideration of the number of parasites present at each replication cycle prior to treatment in each well/animal/individual. Thus, this assumption of independence is used as it allows the same methodology to be used across all experimental systems and removes the need to separately estimate other quantities accurately, such as the inoculum and parasite multiplication rate (PMR) in each well/animal/individual.

For the in vitro regrowth data using endemic donor cells, we used the same approach as for the NSG and VIS datasets. To compute the number of parasites at the time of treatment, we assumed that each culture had a volume of 6 mL, a hematocrit level of 4%, and a mean human RBC volume of 90 fL (as described above). For the estimated frequency of resistant mutants in the in vitro regrowth of 3D7 parasites in culture with endemic donor RBCs, we had parasitemia data for 9 out of the 52 cultures. Thus, we assumed that the number of parasites at the time of treatment for each of the 52 cultures is the mean number of parasites from these 9 cultures to be able to estimate

the frequency of resistant mutants at the time of treatment. We assumed (like before) that at least one resistant mutant was present at the time of treatment in each of the 2 isolates (out of 52 isolates) with resistant mutants.

Estimation of the frequency of mutants allowing for false negatives

In the above estimation of the frequency of resistant mutants, we assumed that there are no false negatives, i.e. a negative well is assumed to have no resistant mutants. However, it is possible that not all resistant parasites are detected, and some wells are falsely categorized as negative wells without resistant parasites. This could be due to, for example, stochastic elimination of a low number of resistant parasites during outgrowth or detection limits of the assay and sequencing for resistant mutants. Thus, to study the importance of assuming that there are no false negatives, we also estimated the frequency of resistant mutants assuming that 5% of wells with at least one resistant parasite are false negatives[7] and 95% of wells with at least one resistant parasite are correctly identified as positive wells. Thus, the probability of a positive well is given by

$$P(\text{positive well}) = 0.95 \times P(\geq 1 \text{ resistant parasite}) = 0.95 \times \left(1 - B(0 \mid n, f)\right), \qquad (5)$$

where *B* denotes the binomial distribution, *n* denotes the number of parasites at treatment, and *f* denotes the fraction of resistant parasites. Using this probability for a positive well, we estimated the fraction of resistant parasites in the different data sets as outlined above and compared the estimate with our previous estimate wherein we assumed that there were no false negative wells (Supplementary Table 7).

Deterministic model for the frequency of resistant mutants

We first developed a deterministic mathematical model of the emergence of resistant parasites (*r*) in a population of parasites sensitive to cabamiquine (*s*). The model includes growth of sensitive parasites with PMR *p*, growth of resistant parasites with PMR p_r, and a mutation rate *m* for resistance mutations. In generation *n*, the number of sensitive parasites and resistant parasites was then described by:

$$s(n) = p\, s(n-1) - m\, s(n-1), \quad s(0) = s_0, \qquad (6)$$

$$r(n) = p_r\, r(n-1) + m\, s(n-1), \quad r(0) = r_0.$$

This model assumes that there is a constant rate at which resistant mutants are generated and that other mutations, such as deleterious mutations or mutation from resistant back to sensitive parasites can be neglected (this may, e.g. be due to deleterious mutations affecting both sensitive and resistant parasites similarly or because mutations back to being sensitive to cabamiquine are very rare). We used a mutation rate of 1.05×10^{-9} base pair substitutions per generation and base pair, i.e. 1 resistant mutant emerging from a parent population of 9.5×10^{8} parasites. This mutation rate is the median of four reported mutation rates for the 3D7 strain of *P. falciparum* reported (Supplementary Table 10)[8–11]. This model parameterization was chosen so that the parameters *p* and *m* approximate, as closely as possible, the parameters of PMR and mutation rate as they are measured in the literature (i.e. the PMR is estimates as the ratio of total parasites between generations and the mutation rate is estimated per parent population). We assumed that there were 11 different substitutions that lead to resistance (Supplementary Table 1, Supplementary Fig. 1A for varying numbers of resistance mutations). In this model, we also assumed that all resistant parasites had the same PMR p_r and thus had the same fitness cost for resistance.

The model equations can be solved explicitly for generation $n \geq 1$:

$$s(n) = s_0 (p-m)^n,$$
$$r(n) = s_0 \frac{m((p-m)^n - p_r)}{p - m - p_r} + r_0 p_r^n. \qquad (7)$$

The quantity of interest in our model of emergence of resistant parasites is the fraction of resistant parasites:

$$\frac{r(n)}{s(n)+r(n)} = \frac{m\left(1 - \left(\frac{p_r}{p-m}\right)^n\right) s_0 + \left(\frac{p_r}{p-m}\right)^n (p-m-p_r) r_0}{(p-m-p_r) s_0 + m\left(1 - \left(\frac{p_r}{p-m}\right)^n\right) s_0 + \left(\frac{p_r}{p-m}\right)^n (p-m-p_r) r_0}. \qquad (8)$$

Thus, we find that the equilibrium fraction of resistant parasites can be calculated as follows:

$$\frac{r(n)}{s(n)+r(n)} \xrightarrow{n\to\infty} \frac{m}{p-p_r} \quad \text{if } \left|\frac{p_r}{p-m}\right| < 1. \qquad (9)$$

Since the mutation rate *m* is several orders of magnitude smaller than the PMR of sensitive parasites (*p*) and the PMR of resistant mutants (p_r) is smaller than the PMR of sensitive parasites (*p*), the condition for convergence to the equilibrium holds for realistic parameter values.

For the fitness cost of resistant parasites, we assumed that the PMR was decreased by *x* percent:

$$p_r = \frac{100 - x}{100} \times p. \tag{10}$$

Considering that several different mutations that confer resistance could occur, we multiplied the mutation rate with the number of possible resistance mutations (n_{res}) and obtained the following equilibrium frequency of resistant parasites:

$$\frac{m \times n_{res}}{p \times x/100} \tag{11}$$

or, equivalently, we expected to find (on average) one resistant parasite per

$$\frac{p \times x/100}{m \times n_{res}} \tag{12}$$

parasites.

Stochastic simulations of the different experimental settings

We used a stochastic model (see Methods in the manuscript) to simulate the different experimental settings (minimum inoculum of resistance [MIR], in vitro regrowth, NSG, and VIS). To this end, we assumed that parasites from a common stock were used for inoculation, and that this stock of parasites had undergone sufficient rounds of replication to have reached the equilibrium frequency of resistant parasites. The equilibrium fraction of resistant parasites was computed as described above using the pre-inoculation PMR, fitness cost of resistance, mutation rate, and number of resistance mutations detailed below (Parameters of the models section). We then sampled the experiment-specific number of parasites from this stock of parasites that were simulated by sampling from a binomial distribution, wherein the number of trials was the number of parasites, and the success probability was the equilibrium fraction of resistant parasites. After inoculation, the experiment was simulated as described in the Methods section in the manuscript with the experiment-specific pre-treatment PMR and number of generations. If an experimental setting included repeated cultures or

multiple hosts, different inocula, different PMRs, or different numbers of generations from inoculation to treatment, then we simulated each culture or host was simulated with its experiment-specific parameters (Supplementary Table 8).

For each simulated experiment, we estimated the frequency of resistant parasites using the same method as for the experimental data. Thus, if a simulated culture or host contained at least one resistant parasite, then we considered this culture or host was considered positive. The frequency of resistant parasites was then estimated using the number of parasites at treatment in each culture or host and the information regarding the presence or absence of resistant parasites (Supplementary Table 9).

Each experiment was simulated 100,000 times and we report the median, 2.5th, and 97.5th percentiles of the estimated frequencies of resistant parasites (squares and dashed lines in Supplementary Fig. 4A and Supplementary Fig. 1).

Parameters of the models

The parameters of the deterministic and stochastic models are the PMR, the fitness cost for resistant parasites, the number of resistance mutations, and the mutation rate. Unless otherwise specified, we used the following values for these parameters:

- **PMR**: We assumed that on average, each parasite had four replicating progeny that were in the range of PMRs of the different experiments (Supplementary Table 8). For the sensitivity analysis (Supplementary Fig. 1), we considered PMRs of 1.2, 2, 4, 5, and 6.

  For the simulations of experiments using the stochastic model, we used this PMR as the pre-inoculation PMR, i.e. the PMR of the stock of parasites that influences the equilibrium frequency of resistant parasites. After inoculation, parasites were simulated to replicate with the experiment-specific pre-treatment PMR (see below).
- **Fitness cost for resistant parasites**: We assumed that all resistant parasites had a 7% reduction in their PMRs compared with sensitive parasites. This is consistent with a fitness cost per generation of 3%-11% that was observed for E134D, L755F, and Y186N (Supplementary Fig. 2). For the sensitivity analysis, we considered fitness costs of 3%, 5%, 7%, 9%, or 11% reduction in the PMR.
- **Number of resistance mutations**: Since we identified 11 amino acid positions for resistance mutations (Supplementary Table 1), we assumed that there were

11 base pair positions for resistance mutations. In the sensitivity analysis, we used varying numbers of resistance mutations: 5, 11, 20, 30, or 40.

- **Mutation rate**: The mutation rate was 1.05×10^{-9} base pair substitutions per generation and base pair which is the median of the mutation rates reported in four different studies (Supplementary Table 10). For the sensitivity analysis, we selected lower and upper bounds that included the reported mutation rates and intermediate (on a $\log_{10}$-scale) parameter values, i.e. 1.0×10^{-10}, 3.2×10^{-10}, 1.05×10^{-9}, 5.6×10^{-9}, and 3.0×10^{-8} base pair substitutions per generation and base pair.

We also used the stochastic model to simulate the different experiments using experiment-specific parameters. These experiment-specific parameter values are summarized in Supplementary Table 8 and described here:

- **Inoculation size**: The inoculation size, i.e. the initial number of parasites, of the MIR experiments is given in Supplementary Table 2. The inoculum size was 2.0×10^{7} parasites for the NSG experiment[2, 3] and 2,800 for the VIS experiment[6]. For the regrowth in vitro experiments, we used the average number of parasites at the first time point after inoculation as the inoculation size.
- **Number of cultures or hosts**: The numbers of cultures with different inoculum sizes, pre-treatment PMRs, or generations to treatment were extracted from Supplementary Table 2 and previous studies[2,3,6].
- **Generations to treatment**: We extracted the number of days between inoculation (or the first time point after inoculation for which the parasitemia level was known) and treatment from the study references[2,3,6] and data. Assuming a life-cycle duration of 40 h for 3D7 parasites[12–14] and 48 hours for field isolates, we computed the number of generations to treatment (n_{gen}):

$$n_{gen} = \text{round}\left(\frac{\text{days from inoculation to treatment} \times 24\text{ h}}{\text{life cycle duration [h]}}\right). \quad (13)$$

hNote that for the MIR experiments, treatment was administered directly after inoculation. Thus, we assumed that no replication occurred between inoculation and drug treatment.

- **Pre-treatment PMR**: After inoculation, parasites replicate and the average number of progeny is the pre-treatment PMR. We computed the pre-treatment

PMR using the average number of parasites at inoculation and treatment and the number of generations as follows:

$$\text{PMR}_{\text{pre-treatment}} = \text{round}\left(\sqrt[n_{gen}]{\frac{\text{mean number of parasites at treatment}}{\text{mean number of parasites at inoculation}}}\right), \tag{14}$$

where n_{gen} is the number of generations from inoculation to treatment.

These experiment-specific parameter values were used for all simulations of experiments and were not varied for the sensitivity analysis (Supplementary Fig. 1).

Probability of emergence of resistant mutants only post treatment

We computed the probability of resistant mutants emerging only after treatment (meaning that no resistant mutants should have emerged until treatment, but they should emerge only after drug treatment) and compared it with the probability that resistant mutants were already present at the time of treatment. We used a mutation rate (*m*) of 1.05×10^{-9} base pair substitutions per generation and base pair (Supplementary Table 10) and 11 possible mutations conferring resistance (n_{res}, Supplementary Table 1).

For the probability that resistant mutants were present at the time of treatment, we used the estimated fraction of resistant parasites estimated from all data combined, i.e. a fraction of 2.33×10^{-9} or 1 resistant mutant per 4.3×10^{8} parasites. Thus, we obtained the probability of resistant mutant emergence, in the same way as we constructed the likelihood function for the estimation of the frequency of mutants, as follows:

$$P(\text{present at treatment}) = 1 - B(0|n, f) \tag{15}$$

where *n* is the number of parasites at the time of treatment and *f* is the fraction of resistant parasites estimated from the data.

If there are *n* parasites that replicate after treatment, then the probability that one of their progeny is a mutant was calculated as follows:

$$P(\text{emerging after treatment | no resistant mutants at treatment}) = 1 - B(0|n, m \times n_{res}). \tag{16}$$

If there are p_0 parasites at the time of treatment and only a fraction λ of parasites can replicate after drug treatment, then there are $\lambda \times p_0$ replicating in the first replication cycle after drug treatment. These parasites have $\lambda \times p_0 \times \text{PMR}$ progeny of which

$\lambda^2 \times p_0 \times \text{PMR}$ can replicate in the second generation. Similarly, the number of parasites replicating after drug treatment can be computed as follows:

$$\lambda p_0 + \lambda^2 p_0 \text{PMR} + \lambda^3 p_0 \text{PMR}^2 + \lambda^4 p_0 \text{PMR}^3 + \cdots \\ = \lambda p_0 \times (1 + \lambda \text{PMR} + (\lambda \text{PMR})^2 + \cdots) \tag{17}$$

We assumed that drug treatment reduces the PMR to ≤1 and defined the parasite reduction ratio (PRR) as follows: $\text{PRR} = \lambda \times \text{PMR}$ (with $\text{PRR} < 1$ by assumption). Thus, an upper bound for the number of parasites replicating after drug treatment was computed as follows:

$$\lambda p_0 \times \sum_{k=0}^{\infty} \text{PRR}^k = \lambda p_0 \times \frac{1}{1 - \text{PRR}} \leq p_0 \times \frac{\text{PRR}}{1 - \text{PRR}} \leq \left\lceil p_0 \times \frac{\text{PRR}}{1 - \text{PRR}} \right\rceil, \tag{18}$$

where we assumed that $\text{PMR} \geq 1$ and denote by $\lceil . \rceil$ the ceiling function. Thus, we estimated that if treatment reduces the PMR below 1, then

$$P(\text{emerging after treatment | no resistant mutants at treatment}) \\ \leq 1 - B\left(0 \,\middle|\, \left\lceil p_0 \times \frac{\text{PRR}}{1 - \text{PRR}} \right\rceil, m \times n_{res}\right). \tag{19}$$

The probability that resistant mutants emerge only after treatment is given as the product of the probability of resistant parasites emerging after treatment (considering that there are no resistant mutants at the time of treatment) and the probability that there are no resistant mutants at the time of treatment:

$$P(\text{emergence only after treatment}) = \\ = P(\text{emerging after treatment | no resistant mutants at treatment}) \\ \times P(\text{no resistant mutants at treatment}) = \\ = \left(1 - B\left(0 \,\middle|\, \left\lceil p_0 \times \frac{\text{PRR}}{1 - \text{PRR}} \right\rceil, m \times n_{res}\right)\right) \times B(0|n, f). \tag{20}$$

Supplementary Figures

Supplementary Figure1. Estimated frequency of resistant mutants for parameter values. To visualize the sensitivity of the model estimates from the stochastic model based on the parameter values, we estimated the frequency of resistant parasites using different parameter values. We varied the number of resistance mutations (**a**), the mutation rate (**b**), the fitness cost of resistance (**c**), and the pre-inoculation parasite multiplication rate (PMR) (**d**). * For the estimated frequency of resistant parasites in the 3D7 in vitro regrowth, we assumed that the parasite number at the time of treatment for each culture is the mean parasite number from the cultures for which the parasitemia at treatment was known (see Supplementary Materials and Methods). To simulate the different experimental settings, we used the inoculum size, pre-treatment PMR, and time to treatment that is specific to each experimental setting (Supplementary Table 8). In addition to the parameter that is varied in the different panels, the parameters used for these model estimates were fitness cost of 7% reduction in the PMR, 11 amino acid sites where mutations were associated with resistance (Supplementary Table 1), a PMR of 4, and a mutation rate of 1.05×10^{-9} per base pair and generation (the black colored dashed line and square indicate simulations with these parameter values). The confidence bounds for the simulations (horizontal dashed lines) are the 2.5^{th} and 97.5^{th} percentiles of 100,000 simulations. The estimated frequency of resistant parasites from the data is shown for each experimental setting for comparison (black line and point).

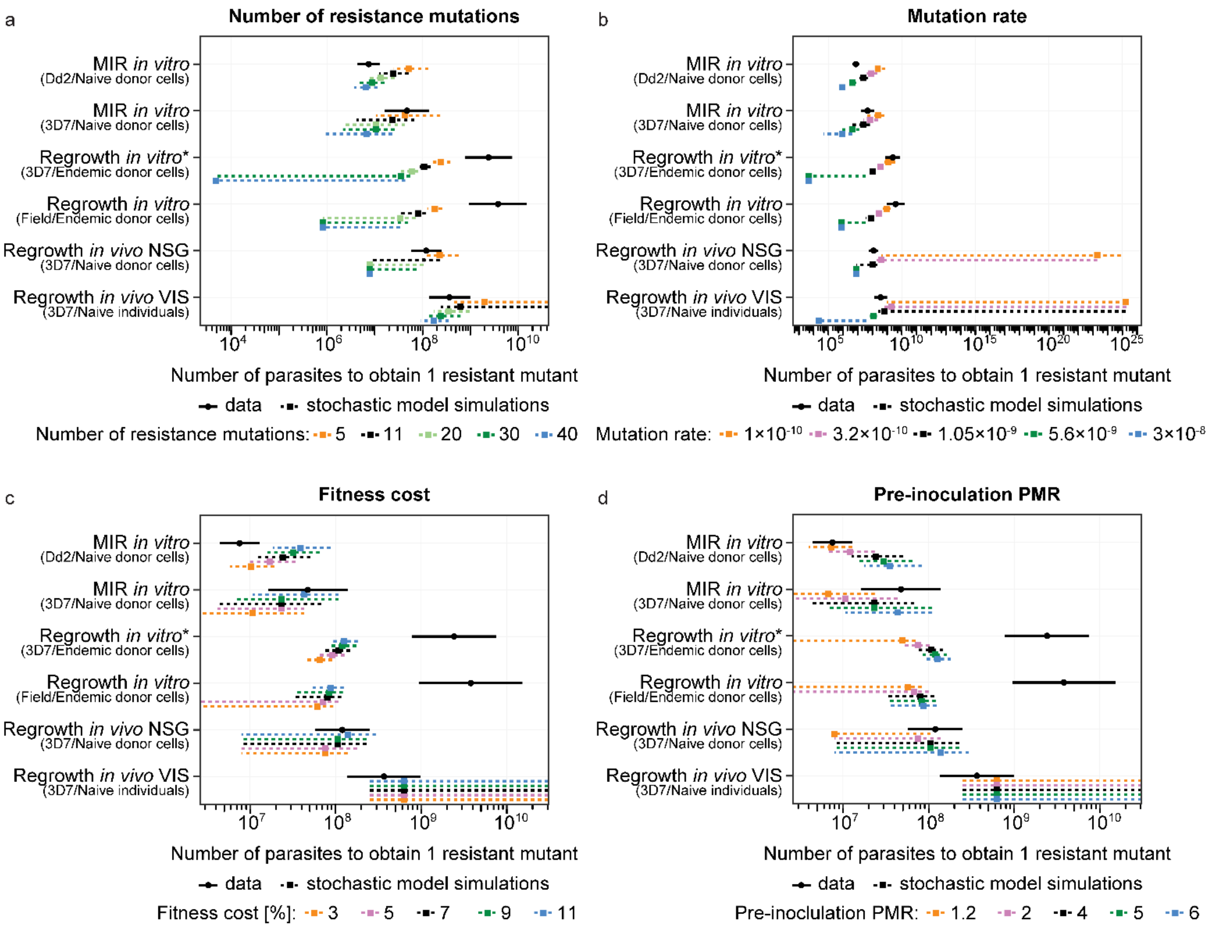
a
Number of resistance mutations
MIR in vitro (Dd2/Naive donor cells)
MIR in vitro (3D7/Naive donor cells)
Regrowth in vitro* (3D7/Endemic donor cells)
Regrowth in vitro (Field/Endemic donor cells)
Regrowth in vivo NSG (3D7/Naive donor cells)
Regrowth in vivo VIS (3D7/Naive individuals)
Number of parasites to obtain 1 resistant mutant
data
stochastic model simulations
Number of resistance mutations: 5 11 20 30 40
b
Mutation rate
Mutation rate: 1×10-10 3.2×10-10 1.05×10-9 5.6×10-9 3×10-8
c
Fitness cost
Fitness cost [%]: 3 5 7 9 11
d
Pre-inoculation PMR
Pre-inoclulation PMR: 1.2 2 4 5 6

Supplementary Figure 2. Fitness cost of cabamiquine-resistant mutants. (a) Fitness cost per generation of three cabamiquine-resistant mutants, relative to the isogenic wild-type (WT) line (mean ± SEM of four independent experiments). **(b)** The EC_{50} of the mutant lines is plotted against the mean fitness cost from (a). For comparison, the mean EC_{50} for WT Dd2 is 0.14 nM. EC_{50} values were obtained from Baragana *et al.*[4]

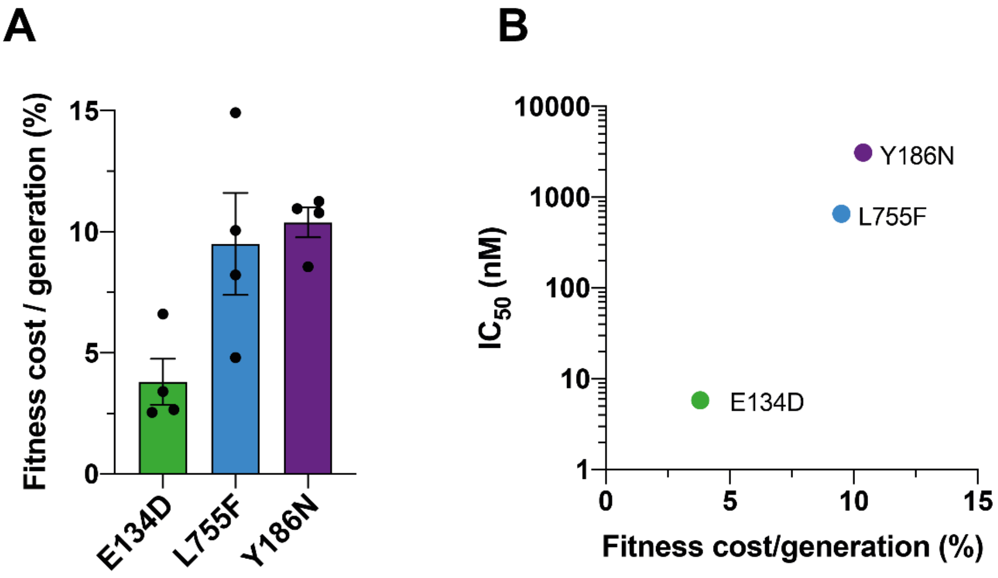

Supplementary Figure 3. Comparison of the deterministic and stochastic models. The frequency of resistant parasites in both the deterministic (blue line) and the stochastic model (black line: median of 100,000 simulations, gray shaded area: 2.5th and 97.5th percentiles of 100,000 simulations) models converges to the equilibrium frequency of resistant parasites. This equilibrium frequency was computed using the deterministic model and was 4.125×10^{-8} or 1 resistant mutant per 2.42×10^{7} parasites (for a PMR of 4, fitness cost of 7% reduction in the PMR, 11 resistance mutations, and a mutation rate of 1.05×10^{-9} mutations per base pair per generation).

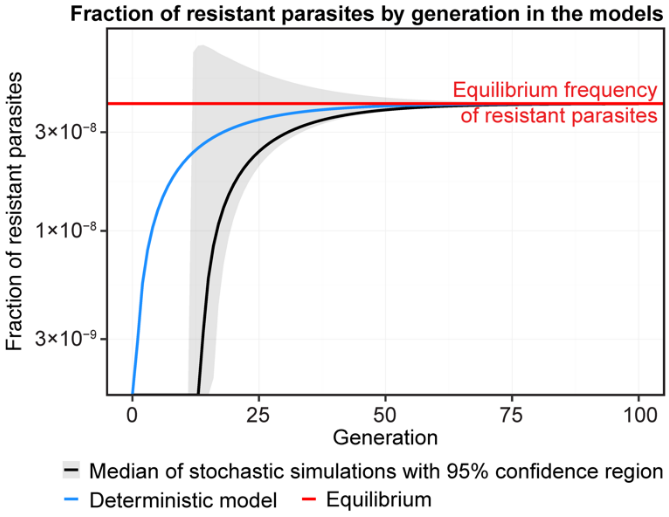

Supplementary Figure 4. Predicted three-dimensional protein structures and potential binding sites close to known mutation sites. (**a**) Protein structure of *Pf*eEF2 predicted using the software tool trRosetta and the location of known mutants (orange). (**b**) Predicted protein structure of *Pf*eEF2 obtained from the Alphafold database and location of known mutants (blue). Close-up of the amino acid residues in proximity to mutants E134 and Y186 on the trRosetta model (**c**) and AlphaFold (**d**) models. (**e**) Binding site detected using SiteMap highlighted by dummy atoms (white spheres) and its surface (orange) close to mutants E134 and Y186 on the trRosetta model. (**f**) Equivalent detected binding site detected using AlphaFold model (blue surface).

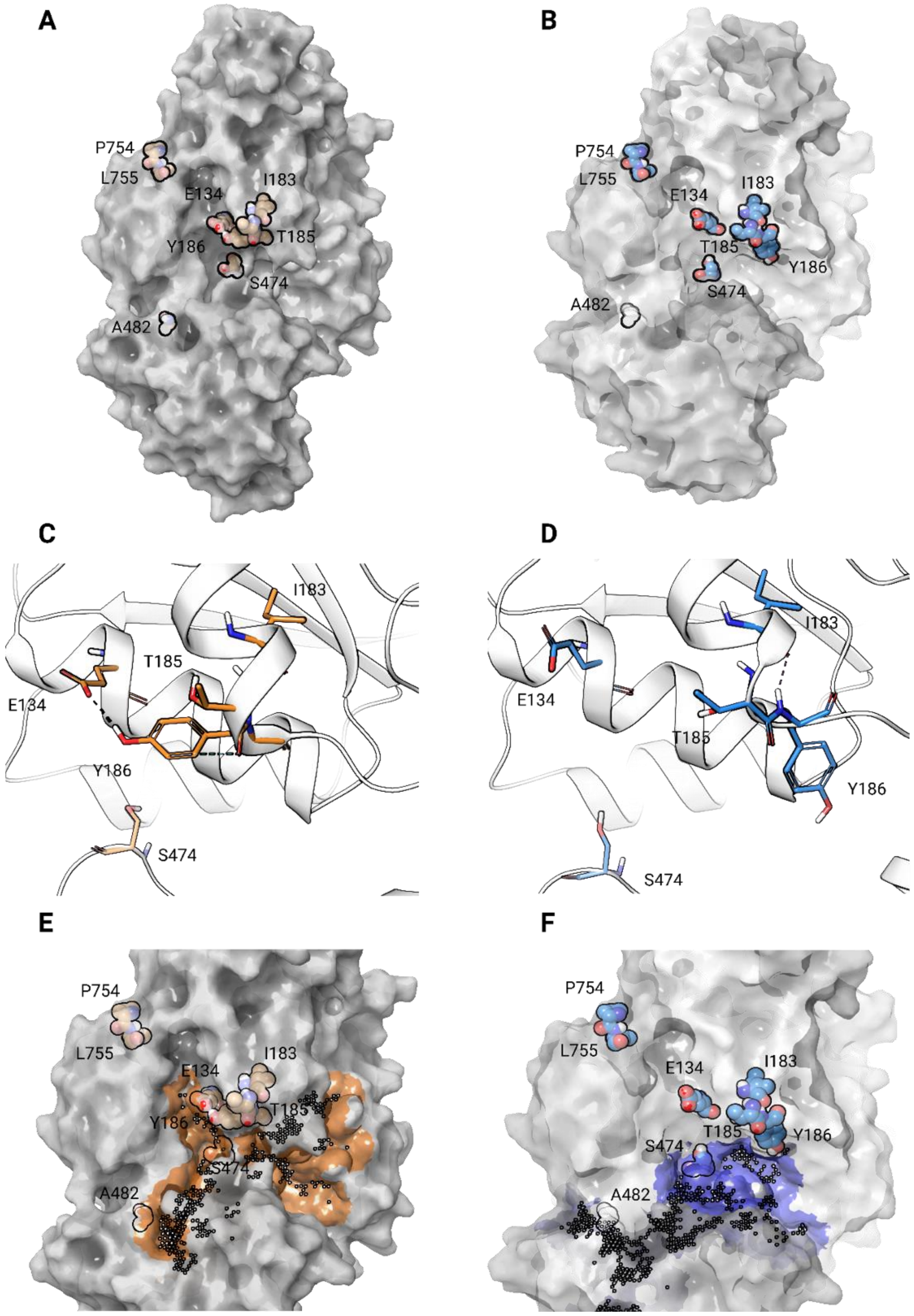
A
P754
L755
E134
I183
T185
Y186
S474
A482
B
P754
L755
E134
I183
T185
Y186
S474
A482
C
I183
T185
E134
Y186
S474
D
I183
E134
T185
Y186
S474
E
P754
L755
E134
I183
T185
Y186
S474
A482
F
P754
L755
E134
I183
T185
Y186
S474
A482

Supplementary Tables

Supplementary Table 1: Summary of cabamiquine resistance-conferring mutations in *Pf*eEF2 selected in vitro or in recrudescent infections in the *P. falciparum* NSG mouse model or in human volunteer infection studies.[3,4,6]

***Pf*eEF2 amino acid position**	**Molecular marker**	***Pf* strain[#]**	**Setting[#]**	**Allele frequency[§]**	**EC$_{50}$ (nM)**	**EC$_{50}$ shift[†]**	**Reference**
134	E134D	Dd2	In vitro	82% (WT: 18%)	6	32×	Baragana, 2015
	E134G	3D7	In vitro	19% (P754S: 81%)<br>20% (P754A: 80%)	59<br>41	211×<br>146×	Baragana, 2015<br>Baragana, 2015
	E134Q	3D7	VIS	100%	ND	ND	McCarthy, 2021
	E134V	3D7_MM*<br>EEF192**	In vitro<br>In vitro	100%<br>100%	109<br>192	191×<br>300×	This study<br>This study
138	Y138C	Pf3D7$^{0087/N9}$	NSG	100%	ND	ND	Rottmann, 2020
182	I182T	Pf3D7$^{0087/N9}$	NSG	100%	137	291×	This study
	I182T	Pf3D7$^{0087/N9}$	NSG	100%	ND	ND	Rottmann, 2020
183	I183T	3D7	In vitro	100%<br>56% (L755F: 44%)	5<br>6	18×<br>21×	Baragana, 2015<br>Baragana, 2015
	I183M	Pf3D7$^{0087/N9}$	NSG	80% (WT: 20%)	2	5×	This study
	I183M	3D7	VIS	100%	ND	ND	McCarthy, 2021
185	T185I	7G8	In vitro	50% (S474R: 50%)	7	29×	Baragana, 2015
186	Y186N	Dd2	In vitro	100%	3,100	16,316×	Baragana, 2015
	Y186C	EEF209**	In vitro	100%	2,626	6,405×	This study
	Y186N	Pf3D7$^{0087/N9}$	NSG	100%<br>Mixed with T753N	ND<br>ND	ND<br>ND	Rottmann, 2020<br>Rottmann, 2020
	Y186N	Pf3D7$^{0087/N9}$	NSG	100%	ND	ND	This study
474	S474R	7G8	In vitro	100%<br>50% (T185I: 50%)	1<br>7	4×<br>29×	Baragana, 2015<br>Baragana, 2015
	S474R	3D7	VIS	100%	ND	ND	McCarthy, 2021
482	A482T	Dd2	In vitro	100%	7	37×	Baragana, 2015
753	T753N	Pf3D7$^{0087/N9}$	NSG	Mixed with WT<br>Mixed with Y186N	ND<br>ND	ND<br>ND	Rottmann, 2020<br>Rottmann, 2020
754	P754S	3D7	In vitro	81% (E134G: 19%)	59	211×	Baragana, 2015
	P754A	3D7	In vitro	80% (E134G: 20%)	41	146×	Baragana, 2015
	P754S	Pf3D7$^{0087/N9}$	NSG	100%	56	140×	This study
	P754L	Pf3D7$^{0087/N9}$	NSG	80% (WT: 20%)	66	140×	This study

	P754A	3D7	VIS	100%	ND	ND	McCarthy, 2021
755	L755F	Dd2	In vitro	100%	660	3,474×	Baragana, 2015
	L755F	3D7	In vitro	44% (I183T: 56%)	6	21×	Baragana, 2015
	L755F	3D7_FS*	In vitro	100%	78	170×	This study
	L755F	$Pf3D7^{0087/N9}$	NSG	Mixed with WT	ND	ND	This study
	L755S	$Pf3D7^{0087/N9}$	NSG	Mixed with WT	ND	ND	This study

[#]Mutants selected either in vitro, in minimum inoculum for resistance (MIR) studies (3D7, 7G8 and Dd2) or in Malian field isolates, in *P. falciparum* ($Pf3D7^{0087/N9}$)-infected humanized mice engrafted with human red blood cells (NSG), or in human volunteer infection (VIS) studies (3D7). *Reference laboratory 3D7 control line propagated in Mali. ***P. falciparum* field isolates (Mali, 2021). [§]For mixed populations, the estimated allele frequencies of wild type (WT) or alternate mutant alleles are indicated in parentheses. [†]EC_{50} shifts were determined for each mutant population compared with its respective WT parental control strain. WT EC_{50} values were as follows: 0.28 nM (3D7), 0.24 nM (7G8), 0.19 nM (Dd2), 0.4 - 0.47 nM ($Pf3D7^{0087/N9}$ in serum), 0.57 nM (3D7_MM), 0.46 nM (3D7_FS), 0.64 nM (EEF192), and 0.41 nM (EEF209). ND, not determined.

Supplementary Table 2. Summary of cabamiquine minimum inoculum of resistance (MIR) assays in Dd2 and 3D7 parasites.

**Parasite strain**	**Dd2**										**3D7**			
EC_{50} (nM)	0.26	0.26	0.34	0.26	0.26	0.33	0.34	0.34	0.26	0.26	0.30	0.30	0.30	0.30
EC_{90} (nM)	0.46	0.46	0.73	0.46	0.46	0.73	0.73	0.73	0.46	0.46	0.45	0.45	0.45	0.45
Inoculum	1×10^4	1×10^5	2×10^5	1×10^6	1×10^7	1×10^7	2×10^7	2×10^7	1×10^8	1×10^9	1×10^6	1×10^7	1×10^8	1×10^9
Selection pressure (nM)	1.25 ($5\times EC_{50}$)	1.25 ($5\times EC_{50}$)	1.5 ($4.4\times EC_{50}$)	1.25 ($5\times EC_{50}$)	1.25 ($5\times EC_{50}$)	1.65 ($5\times EC_{50}$)	1.5 ($4.4\times EC_{50}$)	3.2 ($9.4\times EC_{50}$)	1.25 ($5\times EC_{50}$)	1.25 ($5\times EC_{50}$)	1.5 ($5\times EC_{50}$)	1.5 ($5\times EC_{50}$)	1.5 ($5\times EC_{50}$)	1.5 ($5\times EC_{50}$)
Day of recrudescence	NA (0/3)	NA (0/6)	21 (1/96)	13, 41, 39 (3/9)	13, 13, 15, 20, 22, 22 (6/6)	13 (3/3)	16, 18 (2/2)	13 (1/3)	13, 13, 16 (3/3)	29 (3/3)	NA (0/3)	38 (2/3)	28 (2/3)	20 (3/3)
EC_{50} fold shift (bulk culture)[a]	NA	NA	ND	4,810-5,567×	13×	11-17×	15×	ND	7×	2,315×	NA	14×	99×	19×
MIR values for selections[b]	$\geq3\times10^4$	$\geq6\times10^5$	1.9×10^7	3×10^6	$\leq1\times10^7$	$\leq1\times10^7$	$\leq2\times10^7$	6×10^7	$\leq1\times10^8$	$\leq1\times10^9$	$\geq3\times10^6$	1.5×10^7	1.5×10^8	$\leq1\times10^9$
Reference	Baragana 2015	Baragana 2015	This study	Baragana 2015	Baragana 2015	This study	This study	This study	Baragana 2015	Baragana 2015	Baragana 2015	Baragana 2015	Baragana 2015	Baragana 2015

ND, not determined. NA, not applicable. [a]1-2 recrudescent bulk cultures were phenotyped per selection. [b]MIR is defined as the minimum number of parasites required to achieve a single resistance event and was calculated using the following formula: *(number of parasites in a well)* × *(total number of wells) / (total number of positive wells)*. In this case, we observed three MIRs for Dd2 when tested separately at different selection pressures: 1.9×10^7 when reviewing the 2×10^5 inocula pressured at 4.4× EC_{50} (1 of 96 positive), 3×10^6 when reviewing the 1×10^6 inocula pressured at 5× EC_{50} (3 of 9 positive), and 6×10^7 when reviewing the 2×10^7 inocula pressured at 9.4× EC_{50} (1 of 3 positive).

Supplementary Table 3. Summary of NSG mouse data.

Mouse	Dose [mg/kg]	Parasite recrudescence	Reference
M1_12mpk_2018	12	Mutant	This study*
M2_12mpk_2018	12	Mutant	This study*
M1_30mpk_2018	30	Mutant	This study*
M2_30mpk_2018	30	Mutant	This study*
M_cage1_1_2019	12	Mutant	Rottmann, 2020
M_cage1_2_2019	12	Mutant	Rottmann, 2020
M_cage2_3_2019	12	Mutant	Rottmann, 2020
M_cage2_4_2019	12	Wild-type	Rottmann, 2020
M_cage2_5_2019	12	Wild-type	Rottmann, 2020
M1_2020	12	Mutant	This study*
M2_2020	12	Wild-type	This study*

mpk, mg per kg. * New data obtained following the same protocol as reported by Rottmann, 2020.

Supplementary Table 4. Individual cabamiquine exposure and parasite recrudescence in the VIS[6]. In the 150 mg cohort (n=6) a mean $Cav_{0\text{-}24h}$ of 53 nM was observed with corresponding C_{av}/EC_{99} ratios > 1 (mean [total] = 9; mean [free concentrations] = 3), suggesting sufficient exposures. Three subjects had recrudescing parasites: 1 with wild-type and 2 with *P. falciparum* (Pf) eEF2 mutations being selected at amino acid positions 474 and 183. In the 400 mg cohort (n=8), a mean $Cav_{0\text{-}24h}$ of 217 nM was observed with corresponding C_{av}/EC_{99} ratios of > 10 (mean [total] = 37; mean [free concentrations] = 11), suggesting sufficient exposures. Two subjects had recrudescing infections with parasites bearing *Pf*eEF2 mutations at amino acid positions 134 and 754. In the 800 mg cohort (n=8), a mean $Cav_{0\text{-}24h}$ of 341 nM was observed with corresponding C_{av}/EC_{99} ratios of > 10 (mean [total] = 58; mean [free concentrations] = 18), suggesting sufficient exposures. No recrudescing parasites were detected in this cohort.

Entry	**Dose (mg)**[1]	**$AUC_{0\text{-}24h}$ (total) h*ng/mL**	**$C_{av0\text{-}24h}$ (total) nM**[2]	**$C_{av0\text{-}24h}/EC_{99}$ ratio (total)**[3]	**$C_{av0\text{-}24h}/EC_{99}$ ratio (unbound)**[4]	**$C_{av0\text{-}24h}/EC_{50}$ ratio (unbound)**[4]	**Parasite recrudescence**	***Pf*eEF2 amino acid mutation**
1	150	400	36	6	2	40	Mutant	S474R
2	150	389	35	6	2	39	Wild-type	-
3	150	703	63	11	3	70	No	
4	150	792	71	12	4	79	No	
5	150	683	61	10	3	68	No	
6	150	593	53	9	3	59	Mutant	I183M
7	400	1480	133	23	7	148	No	
8	400	1996	180	30	9	200	No	
9	400	1461	132	22	7	147	Mutant	E134Q
10	400	3085	278	47	15	309	No	
11	400	4111	370	63	20	411	No	
12	400	1758	158	27	8	175	No	
13	400	2412	217	37	11	241	No	
14	400	2970	268	45	14	297	Mutant	P754A
15	800	3791	341	58	18	378	No	
16	800	2212	199	34	10	221	No	
17	800	3719	335	57	18	372	No	
18	800	4569	412	70	22	457	No	
19	800	5907	532	90	28	590	No	
20	800	3438	310	52	16	344	No	
21	800	3679	331	56	17	367	No	
22	800	2929	264	45	14	293	No	

[1]Succinate salt**;** [2]MW 462.6 g/mol; [3]cabamiquine EC_{99} = 5.9 nM (95% confidence interval 4.5–7.6 nM) (n = 39); [4]Human plasma protein binding = 83%, Albumax binding = 45.3%, EC_{50} = 0.28 nM.

Supplementary Table 5. Sensitivity of individual clinical field isolates and 3D7 recrudescent parasites to dihydroartemisinin (DHA). Data are shown as means ± SD from three independent assays.

Parasite strain	DHA EC_{50} (nM)				
	**3D7_FS**	**3D7_MM**	**EEF209**	**EEF192**	**50 other field isolates**
Wild-Type	0.44 ± 0.02	0.65 ± 0.24	0.37 ± 0.09	0.49 ± 0.33	0.76 ± 0.27
Mutant	0.62 ± 0.58	0.54 ± 0.43	0.27 ± 0.11	0.45 ± 0.23	-

Supplementary Table 6. **Overview of the estimated fraction of resistant mutants estimated using different data.**

Data	Fraction of mutants (95% CI)	Number of parasites required to obtain 1 resistant mutant (95% CI)
MIR in vitro (Dd2/Naïve donor cells)	1.33×10^{-7} (7.78×10^{-8} – 2.26×10^{-7})	7.54×10^{6} (4.42×10^{6} – 1.28×10^{7})
MIR in vitro (3D7/Naïve donor cells)	2.11×10^{-8} (7.27×10^{-9} – 6.11×10^{-8})	4.75×10^{7} (1.64×10^{7} – 1.38×10^{8})
Regrowth in vitro* (3D7/Endemic donor cells)	4.14×10^{-10} (1.33×10^{-10} – 1.28×10^{-9})	2.42×10^{9} (7.80×10^{8} – 7.50×10^{9})
Regrowth in vitro (Field/Endemic donor cells)	2.63×10^{-10} (6.59×10^{-11} – 1.05×10^{-9})	3.80×10^{9} (9.50×10^{8} – 1.52×10^{10})
Regrowth in vivo NSG (3D7/Naïve donor cells)	8.35×10^{-9} (4.00×10^{-9} – 1.74×10^{-8})	1.20×10^{8} (5.74×10^{7} – 2.50×10^{8})
Regrowth in vivo VIS (3D7/Naïve individuals)	2.73×10^{-9} (1.01×10^{-9} – 7.33×10^{-9})	3.67×10^{8} (1.36×10^{8} – 9.86×10^{8})

CI, confidence interval

* For the estimated frequency of resistant mutants in the in vitro regrowth of 3D7 parasites in culture with endemic donor red blood cells, we assumed that the number of parasites at the time of treatment for each of the 52 cultures is the mean number of parasites from the 9 cultures for which the parasitemia at treatment was known (see Supplementary Materials and Methods for details).

Supplementary Table 7. Overview of the estimated fraction of resistant mutants estimated using different data and including false negatives. We estimated the fraction of resistant mutants assuming that there were no resistant mutants in negative wells (without false negative wells) or that 5% of wells with resistant mutants would be negative wells (5% false negative wells). A negative well could be a false negative, e.g., due to low numbers of resistant mutants in the well and demographic stochasticity. To compare the estimates with these two assumptions, we report the ratio of estimated frequencies.

Data	Fraction of mutants (95% CI) without false negative wells	Fraction of mutants (95% CI) with 5% false negative wells	Ratio[#]
MIR in vitro (Dd2/Naïve donor cells)	1.33×10^{-7} (7.78×10^{-8} – 2.26×10^{-7})	1.93×10^{-7} (8.39×10^{-8} – 4.46×10^{-7})	1.46
MIR in vitro (3D7/Naïve donor cells)	2.11×10^{-8} (7.27×10^{-9} – 6.11×10^{-8})	9.99×10^{-8} (2.18×10^{-8} – 4.59×10^{-7})	4.74
Regrowth in vitro* (3D7/Endemic donor cells)	4.14×10^{-10} (1.33×10^{-10} – 1.28×10^{-9})	4.36×10^{-10} (1.40×10^{-10} – 1.35×10^{-9})	1.05
Regrowth in vitro (Field/Endemic donor cells)	2.63×10^{-10} (6.59×10^{-11} – 1.05×10^{-9})	2.78×10^{-10} (6.93×10^{-11} – 1.11×10^{-9})	1.05
Regrowth in vivo NSG (3D7/Naïve donor cells)	8.35×10^{-9} (4.00×10^{-9} – 1.74×10^{-8})	1.33×10^{-8} (3.98×10^{-9} – 4.47×10^{-8})	1.60
Regrowth in vivo VIS (3D7/Naïve individuals)	2.73×10^{-9} (1.01×10^{-9} – 7.33×10^{-9})	2.92×10^{-9} (1.07×10^{-9} – 8.01×10^{-9})	1.07

CI, confidence interval

* For the estimated frequency of resistant mutants in the in vitro regrowth of 3D7 parasites in culture with endemic donor red blood cells, we assumed that the number of parasites at the time of treatment for each of the 52 cultures is the mean number of parasites from the 9 cultures for which the parasitaemia at treatment was known (see Supplementary Materials and Methods for details).

[#] Ratio of the fraction of mutants estimated with 5% false negative wells compared with the fraction of mutants without false negative wells.

Supplementary Table 8. Parameters for different data settings. To simulate the different experimental settings using the stochastic model, we used these experiment-specific parameter values.

Data	Inoculum	Number of cultures	Pre-treatment PMR	Days to treatment	Generations to treatment
MIR in vitro<br>(Dd2/Naïve donor cells)	10^4<br>10^5<br>2×10^5<br>10^6<br>10^7<br>2×10^7<br>10^8<br>10^9	3<br>6<br>96<br>9<br>9<br>5<br>3<br>3	0	0	0
MIR in vitro<br>(3D7/Naïve donor cells)	10^6<br>10^7<br>10^8<br>10^9	3<br>3<br>3<br>3	0	0	0
Regrowth in vitro*<br>(3D7/Endemic donor cells)	15,703,704	52	3	4	2
Regrowth in vitro<br>(Field/Endemic donor cells)	18,666,667<br>15,349,593<br>17,066,667<br>16,000,000	5<br>41<br>5<br>1	2<br>2<br>2<br>1	6<br>8<br>10<br>12	3<br>4<br>5<br>6
Regrowth in vivo NSG<br>(3D7/Naïve donor cells)	2.0×10^7	11	3	3	2
Regrowth in vivo VIS<br>(3D7/Naïve individuals)	2,800	22	8	8	5

Supplementary Table 9. Comparison of data estimates and stochastic simulations of experiments.

Data	Fraction of resistant parasites estimated from the data (95% CI)	Fraction of resistant parasites in the simulations of experiments (median, 2.5th and 97.5th percentiles of the simulations)
MIR in vitro (Dd2/Naïve donor cells)	1.33×10^{-7} ($7.78 \times 10^{-8} – 2.26 \times 10^{-7}$)	4.14×10^{-8} ($1.99 \times 10^{-8} – 8.19 \times 10^{-8}$)
MIR in vitro (3D7/Naïve donor cells)	2.11×10^{-8} ($7.27 \times 10^{-9} – 6.11 \times 10^{-8}$)	4.27×10^{-8} ($1.47 \times 10^{-8} – 2.40 \times 10^{-7}$)
Regrowth in vitro* (3D7/Endemic donor cells)	4.14×10^{-10} ($1.33 \times 10^{-10} – 1.28 \times 10^{-9}$)	9.28×10^{-9} ($6.76 \times 10^{-9} – 1.32 \times 10^{-8}$)
Regrowth in vitro (Field/Endemic donor cells)	2.63×10^{-10} ($6.59 \times 10^{-11} – 1.05 \times 10^{-9}$)	1.25×10^{-8} ($8.61 \times 10^{-9} – 2.96 \times 10^{-8}$)
Regrowth in vivo NSG (3D7/Naïve donor cells)	8.35×10^{-9} ($4.00 \times 10^{-9} – 1.74 \times 10^{-8}$)	9.47×10^{-9} ($4.38 \times 10^{-9} – 1.26 \times 10^{-7}$)
Regrowth in vivo VIS (3D7/Naïve individuals)	2.73×10^{-9} ($1.01 \times 10^{-9} – 7.33 \times 10^{-9}$)	1.60×10^{-9} ($6.04 \times 10^{-26} – 4.17 \times 10^{-9}$)

Supplementary Table 10. **Overview of reported mutation rates for the 3D7 and Dd2 strains of *P. falciparum*.**

Reference	Reported mutation rate [per base pair and generation]	
	3D7	Dd2
Bopp, 2013	1.7×10^{-9}	3.2×10^{-9}
Claessens, 2014	4.07×10^{-10}	3.63×10^{-10}
Hamilton, 2017	2.1×10^{-10}	3.2×10^{-10}
Lee, 2016	2.9×10^{-8}	2.2×10^{-8}
Median mutation rate	1.05×10^{-9}	1.78×10^{-9}
Mutation rate range	2.10×10^{-10} - 2.90×10^{-8}	3.63×10^{-10} - 2.2×10^{-8}

Supplementary Table 11. Selected structure-activity relationship of cabamiquine against *P. falciparum* based on data reported by Baragana *et al.*[4] along with docking poses.

**Compounds**	***P. falciparum*** **EC_{50} (nM)[1)]**	***Docking Poses***
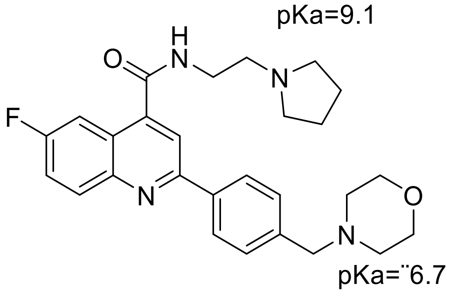 Cabamiquine	1	
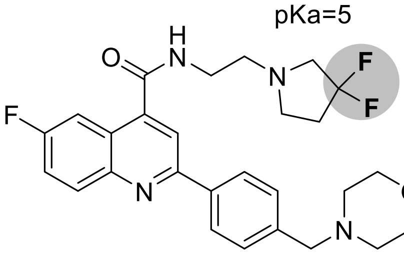 Lower pka results in deprotonated pyrrolidine and no interactions with Glu134	18,000	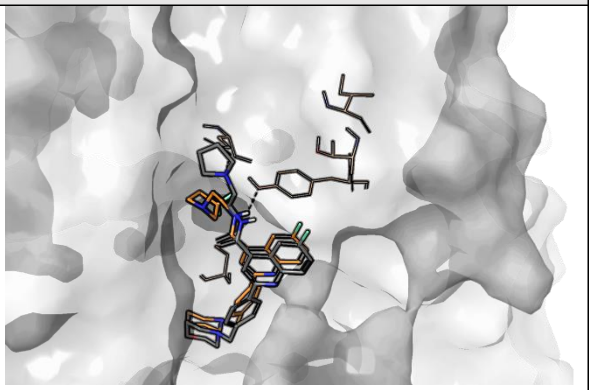
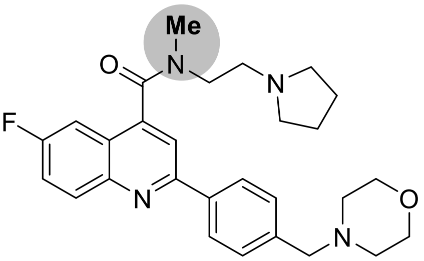 Amide “flipped”, overall structure well aligned	87	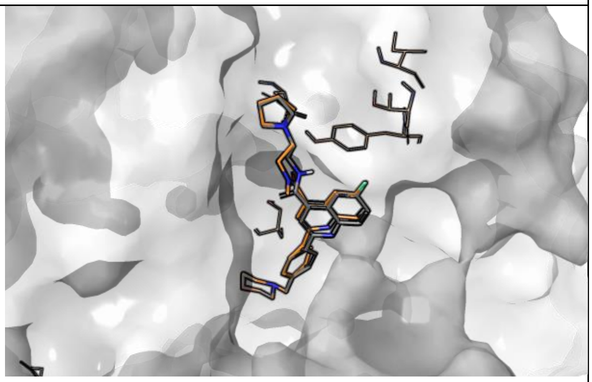
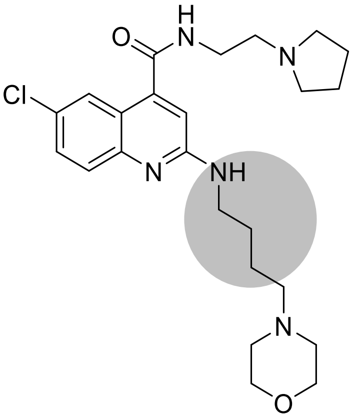 All interactions are conserved, flexible morpholine chain in different direction	4	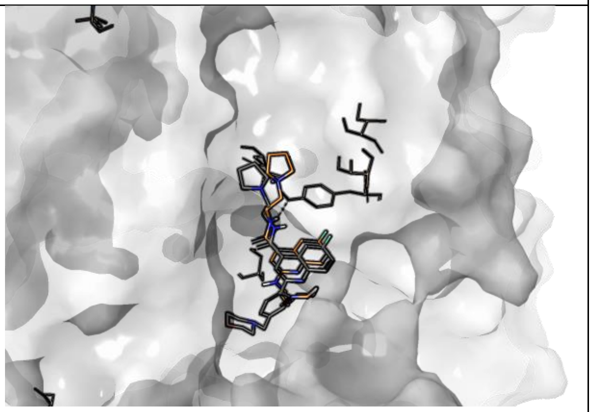

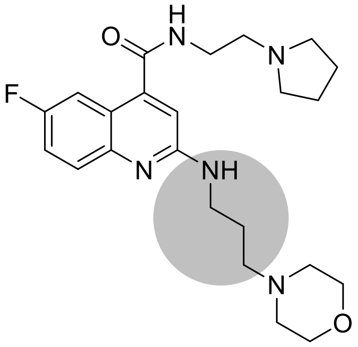<br>All interactions are conserved, flexible 1-carbon shorter morpholine chain in different direction	70	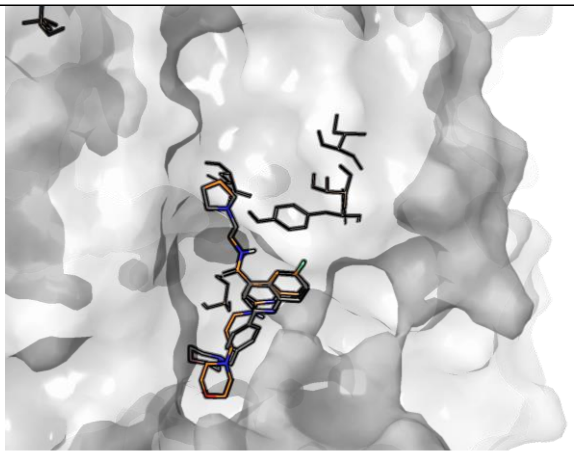
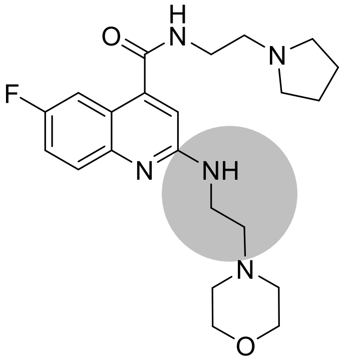<br>All interactions are conserved, flexible 2-carbon morpholine chain in different direction	190	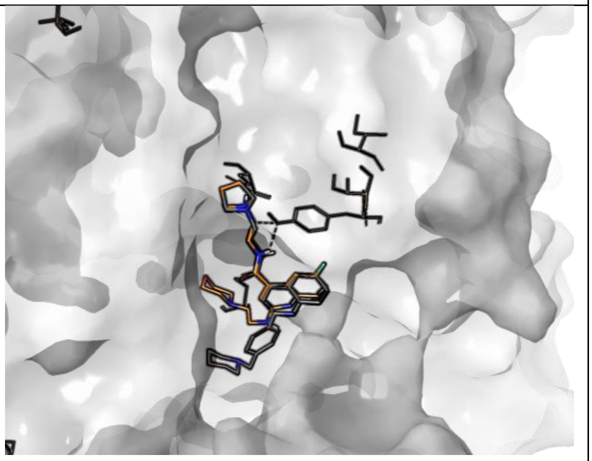
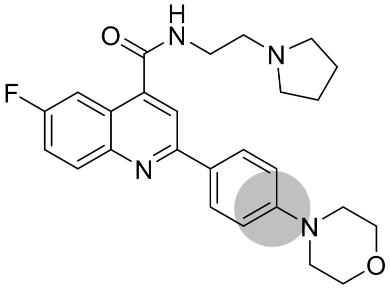<br>All interactions are conserved, rigid 1-carbon shorter morpholine chain in same direction	600	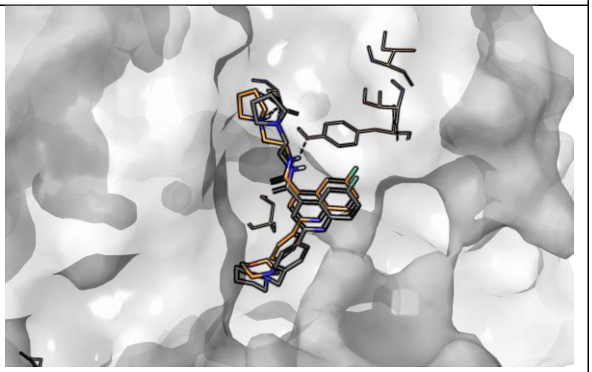

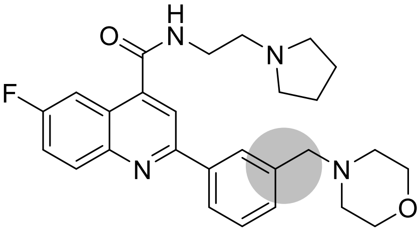 All interactions are conserved, rigid ortho orientated morpholine chain in different direction	11,000	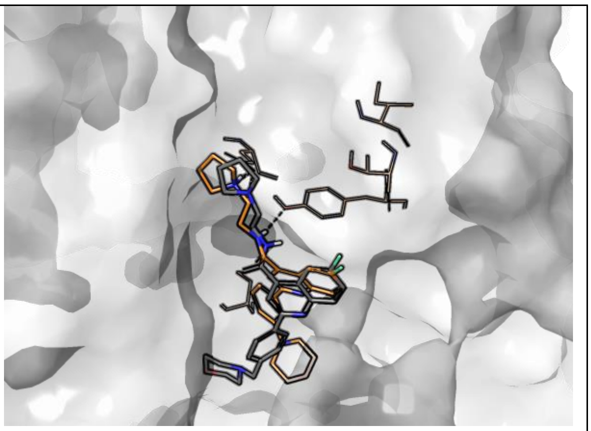

Supplementary Table 12 Primers used for *Pf*eEF2 amplicon sequencing.

Primer	Sequence (5' → 3')	PCR function
p7580	CTTTACGGTAGATCAAGTTCGTG	Outer flank, hemi-nest, sequencing
p7582	AATATTACCGTGTATTTGTATGTAC	Outer flank, sequencing
p7584	CATTTACATATTTGTTGTAGTAGTGG	Hemi-nest, sequencing
p7617	GTCATTATCTCTACATATACTGAC	Sequencing
p7618	CGTAATTGTGATCCTAATGGTCC	Sequencing
p7619	TCATTGTATCTGATCCAGTCGTC	Sequencing
p7620	ATGTTGTTAGTGGTGTGTATGGTG	Sequencing
p7621	CCAGAACCAAAAGATACAGTACC	Sequencing
p7622	AATCTACCCTTATCAGATGTAGG	Sequencing
p7623	TAAACGGTTGTGTTTGTTTGGTG	Sequencing
p7624	GAGTACCTAATTTCTGTTCTTCTG	Sequencing
p7870	TAGCTTCTGGTAAACCTTCAGC	Sequencing

Supplementary References

1. Jimenez-Diaz, M.B., *et al.* Improved murine model of malaria using *Plasmodium falciparum* competent strains and non-myelodepleted NOD-scid IL2Rgammanull mice engrafted with human erythrocytes. *Antimicrob. Agents. Chemother.* **53**, 4533-4536 (2009).

2. Angulo-Barturen, I., *et al.* A murine model of *falciparum*-malaria by in vivo selection of competent strains in non-myelodepleted mice engrafted with human erythrocytes. *PLoS One* **3**, e2252 (2008).

3. Rottmann, M., *et al.* Preclinical antimalarial combination study of M5717, a *Plasmodium falciparum* elongation factor 2 inhibitor, and pyronaridine, a hemozoin formation inhibitor. *Antimicrob. Agents. Chemother.* **64**, e02181-19 (2020).

4. Baragana, B., *et al.* A novel multiple-stage antimalarial agent that inhibits protein synthesis. *Nature* **522**, 315-320 (2015).

5. Stokes, B.H., *et al. Plasmodium falciparum* K13 mutations in Africa and Asia impact artemisinin resistance and parasite fitness. *Elife* **10**, e66277 (2021).

6. McCarthy, J.S., *et al.* Safety, pharmacokinetics, and antimalarial activity of the novel *Plasmodium* eukaryotic translation elongation factor 2 inhibitor M5717: a first-in-human, randomised, placebo-controlled, double-blind, single ascending dose study and volunteer infection study. *Lancet. Infect. Dis.* **21**, 1713-1724 (2021).

7. Alexander, H.K., MacLean, R.C. Stochastic bacterial population dynamics restrict the establishment of antibiotic resistance from single cells. *Proc. Natl. Acad. Sci. U. S. A.* **117**, 19455-19464 (2020).

8. Bopp, S.E., *et al.* Mitotic evolution of *Plasmodium falciparum* shows a stable core genome but recombination in antigen families. *PLoS Genet.* **9**, e1003293 (2013).

9. Claessens, A., *et al.* Generation of antigenic diversity in *Plasmodium falciparum* by structured rearrangement of Var genes during mitosis. *PLoS Genet.* **10**, e1004812 (2014).

10. Hamilton, W.L., *et al.* Extreme mutation bias and high AT content in *Plasmodium falciparum*. *Nucleic Acids Res* **45**, 1889-1901 (2017).

11. Lee, A.H., Fidock, D.A. Evidence of a mild mutator phenotype in Cambodian *Plasmodium falciparum* malaria parasites. *PLoS One* **11**, e0154166 (2016).

12. Khoury, D.S., Cromer, D., Mohrle, J.J., McCarthy, J.S., & Davenport, M.P. Defining the effectiveness of antimalarial chemotherapy: Investigation of the lag in parasite clearance following drug administration. *J. Infect. Dis.* **214**, 753-761 (2016).

13. Douglas, A.D.*, et al.* Comparison of modeling methods to determine liver-to-blood inocula and parasite multiplication rates during controlled human malaria infection. *J. Infect. Dis.* **208**, 340-345 (2013).

14. Wockner, L.F.*, et al.* Growth rate of *Plasmodium falciparum*: Analysis of parasite growth data from malaria volunteer infection studies. *J. Infect. Dis.* **221**, 963-972 (2020).
